# Ocular Autonomic Nervous System: An Update from Anatomy to Physiological Functions

**DOI:** 10.3390/vision6010006

**Published:** 2022-01-14

**Authors:** Feipeng Wu, Yin Zhao, Hong Zhang

**Affiliations:** Department of Ophthalmology, Tongji Hospital, Tongji Medical College, Huazhong University of Science and Technology, Wuhan 430030, China; 15927161371@163.com (F.W.); tjyksys@163.com (H.Z.)

**Keywords:** autonomic nervous system, eye, anatomy, pupil light reflex, choroidal blood flow, aqueous humor

## Abstract

The autonomic nervous system (ANS) confers neural control of the entire body, mainly through the sympathetic and parasympathetic nerves. Several studies have observed that the physiological functions of the eye (pupil size, lens accommodation, ocular circulation, and intraocular pressure regulation) are precisely regulated by the ANS. Almost all parts of the eye have autonomic innervation for the regulation of local homeostasis through synergy and antagonism. With the advent of new research methods, novel anatomical characteristics and numerous physiological processes have been elucidated. Herein, we summarize the anatomical and physiological functions of the ANS in the eye within the context of its intrinsic connections. This review provides novel insights into ocular studies.

## 1. Introduction

The ANS plays a major role in maintaining the physiological integrity of the entire body in response to environmental alterations. Similar to the other organs in the body, the majority of intraocular muscles of the eyes are dominantly controlled by the ANS. Moreover, the pupillary light reflex (PLR) has been widely used for the detection of ANS dysfunction [1].

**Figure 1:**
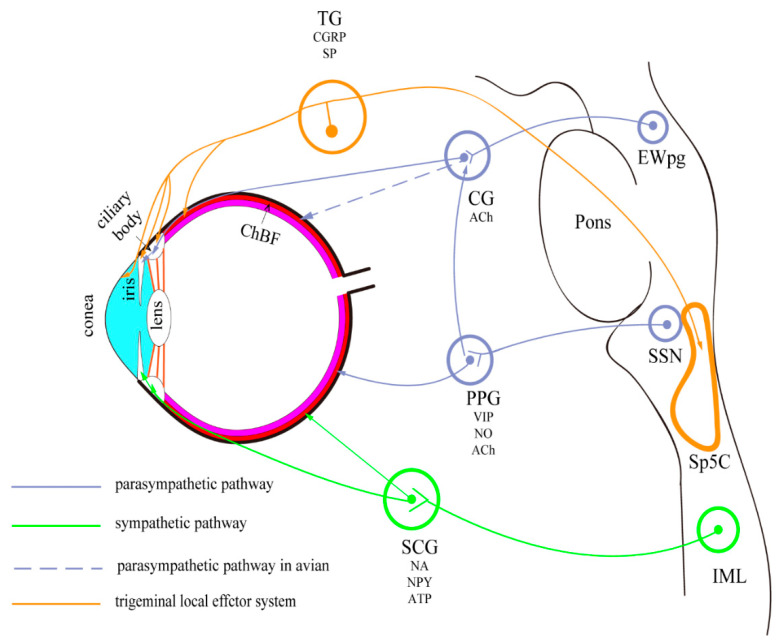
Autonomic pathways in mammals and avian. The encircled areas represent nuclei and ganglia. There are three autonomic pathways: (1) EW→CG→targets (parasympathetic pathway); (2) SSN→PPG→targets (parasympathetic pathway); (3) IML→SCG→targets (sympathetic pathway). Trigeminal nerve branches are also present. CG—ciliary ganglion; ChBF—choroidal blood flow; EWpg—nucleus of Edinger-Westphal; preganglionic division; IML—intermediolateral column; PPG—pterygopalatine ganglion; SCG—superior cervical ganglion; SSN—superior salivatory nucleus; TG—trigeminal ganglion.

Briefly, the eyes are innervated by the sympathetic, parasympathetic, and trigeminal sensory nerve fibers (Figure 1). The preganglionic fibers of the parasympathetic nerve are derived from the Edinger-Westphal preganglionic (EWpg) and superior salivatory (SSN) nuclei [2]. The sympathetic innervation of the eye is derived from the preganglionic neurons located in the C8-T2 segments of the spinal cord, ascending to the superior cervical ganglion [3]. These autonomic neurons have anatomical differences in various species, which will be discussed in this review.

Numerous studies have identified newer autonomic pathways and peptides secreted by these nerve fibers in various species. These studies have helped shape and enrich the knowledge about the eye under different physiological and pathological conditions. The historical and current knowledge of the anatomy and physiological processes of the ANS in the eye are summarized in this review.

## 2. The Autonomic Pathways in the Eye

### 2.1. Parasympathetic Pathways (Edinger-Westphal Nucleus–Ciliary Ganglion)

From around 21 weeks of gestation in humans, the Edinger-Westphal nucleus (EWn) forms a unique and complex 3D structure consisting of 2–3 parts [4]. In primates, the EWn lies immediately dorsal to the somatic subdivisions of the oculomotor nuclear complex, which contains the neurons that form the oculomotor nerve within the midbrain. These nerves travel to the bilateral ciliary ganglion (CG) via the oculomotor root of the ciliary ganglion; the postganglionic fibers enter the eyeball through the short ciliary nerve, along with other sympathetic postganglionic and trigeminal sensory fibers. Within the eyeball, they distribute in the pupillary sphincter and ciliary muscles where they exert their functions. Since the first description of EWn in the literature, it has been attributed with the function of providing parasympathetic control to the eye. This is true in monkeys and birds [5,6,7]. However, in mice, rats, ferrets, cats, and humans, the parasympathetic preganglionic neurons projecting to the CG do not accurately correspond with the reported location in the cytoarchitecturally defined EW; these neurons are scattered outside the EWn and in the perioculomotor region, ventral to the oculomotor nucleus [3,8]. This difference in localization has created confusion. It has been suggested that “the cholinergic, preganglionic neurons supplying the CG are termed the Edinger-Westphal preganglionic (EWpg) population. The other part, the centrally projecting peptidergic neurons, is termed the Edinger-Westphal centrally projecting (EWcp) population” [9]. In mice, rats, ferrets, cats, and humans, the EWcp, which was previously defined cytoarchitecturally, has been shown to contain peptidergic neurons that express urocortin-1 and project centrally within the brain, instead of cholinergic neurons [8,10]. Further research on the ultrastructure of macaque EWpg motoneurons suggested the presence of multiple inputs from different types of neuronal terminal. Using neural circuit tracing methods, the motoneurons in the macaque EWpg have been observed to be organized into a single column that runs longitudinally and dorsally to the oculomotor nucleus [11].

The CG, as a transfer station for the parasympathetic nerve behind the eye, is mainly involved in ocular accommodation and pupil constriction. Around 3% of the postganglionic neurons from CG in macaques were showed heading toward the sphincter pupillae, and in cats, this portion increases to 10–15% [12,13]. The CG is a small red-gray quadrangular plate, approximately 3 mm long, situated near the apex of the orbit and specifically varies according to the position of the optic nerves [14,15]. Additionally, known as the ciliary nerves, these nerves include three roots (sympathetic, parasympathetic, and sensory fibers), attached to the CG. Moreover, from the anterior border, ciliary nerves give rise to 8–10 branches, arranged in 2 bundles [14,16]. In humans, numerous substance P (SP)- and calcitonin gene-related, peptide (CGRP)-immunoreactive, non-varicose nerve fibers are connected with the ganglion cells and nerve trunks that enter the ganglia; these findings indicate the input of the trigeminal nerve to CG [17]. Studies that analyzed the macaque CG ultrastructure suggest that this ganglion may also be the site for neuronal processing of the preganglionic input, rather than transmission of the parasympathetic outflow to the eye alone [18]. Immunohistochemical analyses of the gamma-aminobutyric acid (GABA)-synthesizing enzyme, glutamic acid decarboxylase, and choline acetyltransferase (ChAT) have shown that all macaque CG neurons have contact with the ChAT-positive terminals, and that GABA may act as a neuromodulator in controlling the lens or pupil functions [19]. In rats, a few ciliary ganglia and accessory ciliary neurons exhibit VIP immunoreactivity, while, in contrast, in humans and guinea pigs, this immunoreactivity is noted in the uveal ganglion cells [20]. In mammals, neuropeptide Y (NPY) is found in most of the sympathetic ganglion neurons, several parasympathetic ganglion neurons, and in the intramural ganglia of the sympathetic nervous system (SNS). NPY-like immunoreactive (LI) nerve terminals surround 80% of macaque CG cells; however, in macaques, the CG cell somata remain unstained [21]. The size, shape, and topography of, and the various peptides secreted by, CG vary considerably among the species, and findings from animal studies cannot be totally generalized to humans [20,22,23,24,25].

In avians, the CG also innervates the choroid, but there is little evidence for this connection in mammals. Some studies have reported the possibility for such connections, but without any definite evidence [7,26,27,28,29]. The CG consists of two different neuronal populations that are derived from the EWn: the “ciliary neuron”, which innervates the constrictor muscle of the iris and the ciliary body; the “choroidal neuron”, which innervates the choroidal blood vessels [7]. By immunohistochemical methods, the choroidal blood flow of the upper and temporal parts of the eye has been observed to be preferentially affected. In pigeons, adequate blood supply to these parts is needed to maintain high sensitivity and binocular vision [28].

### 2.2. Parasympathetic Pathways (Superior Salivatory Nucleus–Pterygopalatine Ganglion)

The SSN is located approximately dorsolateral to the facial motor nucleus, while the precise location of the SSN in humans has not been identified yet. The preganglionic axons reach the geniculate ganglion as part of the intermediate nerve before separating into two branches: the greater petrosal nerve and the chorda tympani. The greater petrosal nerve contains parasympathetic nerve fibers that extend to the pterygopalatine ganglion (PPG); these fibers control the functions of the lacrimal glands, and the mucous glands of the nose, palate, and pharynx. The branch of the facial nerve, also known the chorda tympani nerve, projects to the submandibular ganglion where it mediates other functions that are not related to the eyes. The cerebral circulation is innervated by the posterior parasympathetic fibers from the PPG as well [30].

The PPG is also known as the sphenopalatine ganglion, Meckel’s ganglion, or the nasal ganglion. Several studies have shown that the PPG is the main source of choroidal parasympathetic input in mammals [31,32,33,34]. The PPG efferent fibers in the choroid secrete the vasodilators VIP and nitric oxide (NO) and appear to be cholinergic in their functions [29]. In addition, a few postganglionic nerve fibers of the PPG have been observed in the short ciliary nerves that regulate choroidal blood flow [35]. In cats, mice, and primates, the lacrimal glands are innervated by the pterygopalatine ganglia, and the terminals of these nerve fibers contain numerous clear vesicles, without any direct synaptic contacts on these structures [36,37,38].

### 2.3. Sympathetic Pathways Innervating the Eye

W.H. Gaskell, in the 1800s, identified the intermediolateral cell column (IML) as the origin of the sympathetic nerve [39]. These sympathetic fibers projecting to the head and globe originate from the C8-T2 segment of the spinal cord and eventually ascend to the superior cervical ganglion (SCG).

The SCG is located at the level of the C2/C3/C4 transverse process, and it is approximately 3–5 cm long and lies proximal to the longus capitis muscle [40]. It radiates along the internal carotid artery in the sympathetic plexus, passes through the cavernous sinus to the orbit, and innervates the pupil dilator [41]. Furthermore, the SCG provides various autonomic inputs to the head and neck organs, including the lacrimal glands and salivary glands, and also regulates choroidal blood flow. Previous studies of the SCG have investigated the applicability of the ratio of the preganglionic fibers to the ganglionic neurons in different species as an indicator of the whole sympathetic nervous system; the ratio ranged from 1:28 for the squirrel monkey ganglion to 1:196 for the human ganglion [42,43,44]. Bundles of sympathetic nerve fibers are found in the trigeminal ganglia and PPG in rats, most of which innervate the iris and ciliary body via the base of the ciliary body [45].

### 2.4. Trigeminal Nerves—A Part of the Somatosensory System

The ophthalmic nerve, a branch of the trigeminal nerve, is mainly responsible for mediating the sensation in the ciliary body, iris, lacrimal gland, conjunctiva, and cornea of the eye. These sensations include touch, pain, and temperature. Though ophthalmic nerve does not belong to autonomic nervous system, this nerve also contains sympathetic nerve fibers that control pupil dilation [46]. Studies using immunohistochemistry and retrograde tracing methods have reported the co-localization of substance P and calcitonin gene-related peptide (CGRP) in the trigeminal ganglion (TG) in mammals [47,48].

## 3. Autonomic Control of the Eye

### 3.1. Cornea

The cornea is one of the most densely innervated parts of the human body with a rich supply of sensory and autonomic nerve fibers [49]. Sensory nerves, mainly originating from the ophthalmic branch of the trigeminal nerve, comprise the majority of the nerves in the cornea. Sympathetic nerves originating from the SCG are found in the corneas of mammals, and their densities vary in different species. Parasympathetic innervation originating from the CG has been also reported in cats and rats [50,51,52].

The corneal innervation was first described in detail in 1951 [53], but the description of human corneal nerves was relatively limited at that time. Recent studies have suggested that nerve bundles enter the peripheral cornea radially and parallel to the corneal surface; thereupon, they lose their perineurium and myelin sheaths at approximately 1 mm from the limbus and about 0.1 nm from the ocular surface. These nerve bundles radiate through the middle third of the corneal stroma and further subdivide to form smaller branches that comprise a moderately dense midstromal plexus and a dense subepithelial plexus (SEP) before finally passing through the anterior elastic layer and entering the corneal epithelium [51,54,55].

These sensory and autonomic nerves also play a key role in maintaining the optimal health of the ocular surface. Corneal nerves can release soluble trophic substances that promote lacrimal gland secretion, induce blinking reflexes, and maintain the integrity of the ocular surface [56]. Substance P (SP) exerts a strong synergistic effect with insulin-like growth factor-1 (IGF-1) or epidermal growth factor to promote corneal epithelial migration, adhesion, and wound closure. Several other neuropeptides also play important roles in the cornea (Table 1) [57,58,59].

### 3.2. Iris

The iris is a circular pigmented membrane located in front of the lens, it divides the cavity between the cornea and lens into an anterior chamber and a posterior chamber.

The iris controls the amount of light entering the eye by adjusting the pupil. The preganglionic parasympathetic axons originating from the EWpg innervate the pupillary sphincter through the short ciliary nerve. These axons act on the muscarinic receptors of the pupillary sphincter, and mediate pupillary constriction. Sympathetic innervation originates from the SCG and provides a reciprocal function via the long ciliary nerves which control pupil dilation [60,61]. The ophthalmic nerve, a branch of the trigeminal nerve, is also involved in the innervation of the iris [62]. Previous studies have suggested that the trigeminal nerve offers sensory transduction and induces substance P-related contractions for mediating protective reflexes [63]. However, recent studies have demonstrated that the trigeminal nerve also affects smooth muscle response, intraocular blood vessels, and immune function by releasing various peptides [60]. Based on these anatomical functions and innervations, pupil evaluation can be a simple and convenient method to detect autonomic disorders and may therefore have potential diagnostic value [64].

The iris of several vertebrate species has rhodopsin, a molecule that enables photomechanical responses (PMR). Rhodopsin enables pupillary constriction in response to light without the need for a central nervous reflex. This process may involve (1) rhodopsin-activated G-protein, (2) phospholipase C, (3) inositol triphosphate, or (4) Ca^2+^ [65,66]. Moreover, PMR can be inhibited by β-adrenergic agonists, but not by α-adrenergic agonists [66,67]. After the application of β-adrenergic agonist to toad sphincter cells, the availability of Ca^2+^ ions for sphincter contraction was found to be altered, followed by pupillary dilation [67]. Generally, the sympathetic and parasympathetic systems work antagonistically to control the contraction and relaxation of the iris muscles. The ratio of innervations from these systems differs among species [60].

### 3.3. Anterior Chamber Angle

The main outflow pathway of aqueous humor (AH) comprises the trabecular meshwork (TM), the endothelial lining of Schlemm’s canal, juxtacanalicular connective tissue, collecting channels, and aqueous veins. Additionally, the outflow resistance of the TM pathway seems to be regulated by the contraction of the scleral spur (SS) cells and ciliary muscles [68]. In almost all species, the SS cells contain SP-positive axons, most of which also immunostain for CGRP [62,69]. In humans, the axons in the TM and SS show immunoreactivity (IR) to SP, CGRP, NPY, VIP, and NOS, while in monkeys, sympathetic SS cell innervations are more frequently observed [70,71,72,73,74]. These cholinergic and nitrergic nerve terminals may induce the contraction and relaxation of TM and SS cells [75]. However, research on human and monkey eyes has shown few TH-positive and VIP-positive nerve fibers, as well as the absence of NPY-positive fibers in SS and TM [75]. Only a small amount of opioid peptidergic innervation has been reported in the anterior eye segment of the eye in rats. SP, CGRP, NPY, and VIP immunoreactivity also occurs in the ciliary process, ciliary muscles, and ciliary blood vessels [27]. A recent study demonstrated the presence of efferent nerve pathways from the hypothalamus to the autonomic innervation in the bilateral anterior chamber [76]. When inflammation occurs, peptide expression in the bilateral anterior chamber is upregulated [77]. The immunolabeling pattern for TM is similar in humans and pigs [78].

Nomura found that approximately one-third of the innervation in the TM is sympathetic in monkeys, and that two-thirds are parasympathetic [79]. In human TM and cultured TM cells, most receptors on the sympathetic nerves appear to be the β2 adrenergic subtype [80,81].

### 3.4. Lacrimal Glands

The lacrimal gland is located in the orbit of the human eye. Lacrimal secretions are vital to the health and maintenance of the cells on the ocular surface (conjunctiva, corneal epithelium). Regulation of lacrimal gland secretion involves the following: (1) stimulation of the sensory nerve on the ocular surface and (2) parasympathetic and sympathetic activation of the lacrimal secretory cells [82]. Mechanonociceptors, polymodal nociceptors, and cold receptor fibers are distributed on the conjunctiva and cornea [83,84]. Stimulation of the corneal polymodal nociceptors causes reflex tear secretion, while mechanonociceptors and cold receptors are less effective in mediating this effect. Interestingly, tear secretion does not increase with increased stimulation of the conjunctival receptors [85].

Tear production is regulated by both the sympathetic and parasympathetic nerves. Generally, sympathetic nerves affect tear secretion via the following two methods: (1) alteration of blood flow [86] and (2) via increased secretion of sympathetic neurotransmitters [87,88]. However, the role of sympathetic nerves in the lacrimal gland remains uncertain. Some studies have suggested that electrostimulation of the SCG alters tear secretion, while other studies report contradictory findings [89,90,91]. The content of the tear secretion remains unaltered even after SCG ablation, indicating that tear secretion is not related to sympathetic postganglionic nerves [92]. Tear secretion is mainly controlled by the parasympathetic nerves [92]. These nerves mediate tear secretion by releasing Ach and activating the M3 muscarinic Ach receptors [93,94]. Moreover, marked reduction in lacrimal gland secretion can be observed in rabbits with parasympathetic nerve lesions [95].

### 3.5. Retina

The neural retina is a layered structure that converts photic illumination into visual information and then transmits this signal to the brain. The retinal circulation and the choroidal circulation, both of which originate from the ophthalmic artery, are responsible for supplying oxygen and nutrition to the retina. Retinal circulation provides a low level of blood flow and a high level of oxygen extraction, contrary to that in choroidal circulation [96,97]. There is limited insufficient evidence of autonomic innervation in the intraocular branch of the central retinal artery (CRA) [98,99]. Instead, retinal circulation is generally considered to be autoregulated by local mechanical and chemical stimulations based on the sensation of the oxygen levels [100,101].

The preocular CRA in humans receives adrenergic and cholinergic nerve fibers, suggesting sympathetic and parasympathetic innervation. However, SP, CGRP, and VIP were not detected in the nerve fibers, indicating a lack of peptidergic innervation [102]. Similarly, immunohistochemical and histochemical studies have confirmed the presence of [95] parasympathetic nerve (NOS/VIP/NADPH-d), sympathetic nerve (TH), and CGRP-positive afferent nerve fibers in the vicinity of the monkey and rat CRA [103,104]. Substance P-positive nerve fibers have also been identified around the CRA in rabbits [105]. Early research on several monkey species has shown that adrenergic innervation is only present posterior to the lamina cribosa in the intraorbital part of the optic nerve [99,106]. Postganglionic nerves of the pterygopalatine ganglion release NO, causing vasodilation of the arterial smooth muscles [107,108]. The sympathetic innervation gradually decreases with increasing age [109], which may lead to significant loss of photoreceptor cells and increased reactivity of the glial cells [110].

### 3.6. Choroid

The choroid makes up the posterior part of the uvea, located between the retina and sclera, and is mainly composed of blood vessels. Neurons in the choroid, which mainly regulate choroidal circulation, are also called choroidal ganglion cells or intrinsic choroidal neurons (ICN) [111,112]. There are approximately 2000 ICNs in each eye. Most of these ICNs are clustered in the temporal and central regions of the submacular area. In contrast, these neurons are largely accumulated at the periphery in rabbits, possibly because rabbits lack the macula [113]. In other species, there are only approximately 500 ICNs, with largely uniform distribution. In different avian species, the number of ICNs varies from less than 500 in quail to more than 6000 in geese. These cells are mainly concentrated at the temporocranial area in Galliformes, while in Anseriformes, they form a belt that extends in the cranionasal to temporocaudal direction [114].

In mammals, the primary input of the parasympathetic nerves originates from the PPG via the facial nerve [33,115]. Although some physiological changes have been noted upon stimulation of the CG, the innervation of the choroid in mammals has not been confirmed to date [116,117,118]. However, in avian, the CG supplies most of the parasympathetic innervation [7], these parasympathetic input increases choroidal blood flow of via vasodilation. On immunohistochemistry, almost all ICGs are stained for nNOS and VIP, half of the cells show immunoreactivity for calretinin, while the individual cells are stained only for just neuropeptide Y (NPY) [34,111]. The choroid contains large amounts of TH(+) and NPY(+) ICNs in the central temporal area [119]. Using immunohistochemistry method, sympathetic innervation was observed derived from the SCG and modulates the NO/VIP/GAL innervation of vascular and non-vascular smooth muscle provided by ICN [120,121]. Such dual innervation balances the increase and decrease in choroid blood flow. Despite aging, the choroidal innervation patterns and neural transmitters remain unaltered to some extent [111]; however, some studies have suggested a decrease in adrenergic fibers and VIP(+) nerve fibers [122,123,124]. SP (+) and CGRP (+) ICNs have been identified in choroidal whole mounts, suggesting choroidal innervation by sensory nerves. Additionally, these ICNs were found to be more concentrated in the temporal and central regions and are thought to be involved in mediating blood flow and vascular architecture [125].

## 4. Physiological Effect

### 4.1. Pupil Adjustment

The size of the pupil varies with the intensity of incident ambient light, which forms the PLR. Generally, pupil dilation and constriction depends on both autonomic innervation and local reflexes [126]. Light incident into one eye can cause constriction of both the eyes, including the unstimulated eye. These phenomena are termed direct and consensual responses. In early studies, the consensual response was thought to be limited to “higher” mammals. However, recent studies have provided more evidence to support that consensual response also occurs in “lower” mammals, and even non-mammals; although, in non-mammalian species, consensual PLR is indistinctive [127,128].

#### 4.1.1. Parasympathetic Pathway

**Figure 2:**
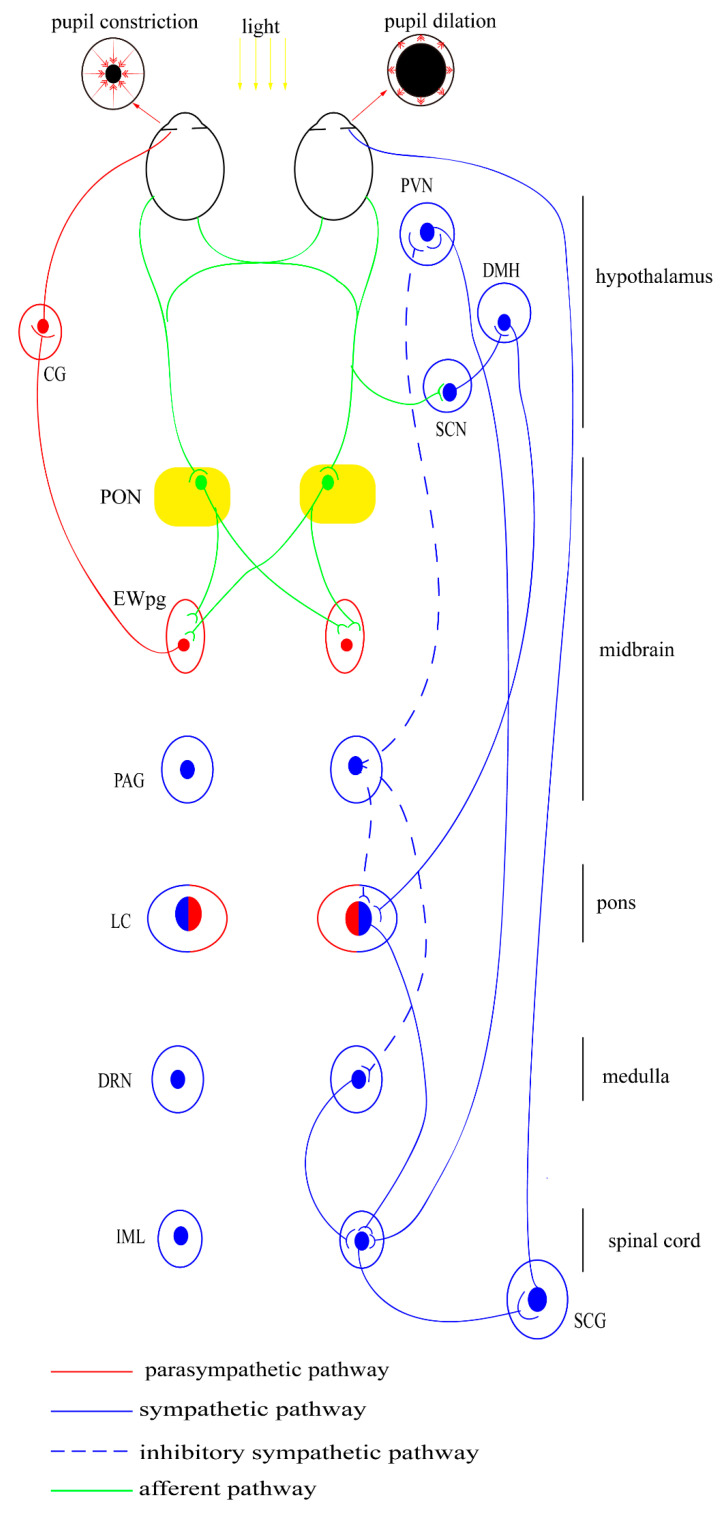
Autonomic pathway of PLR. The encircled areas represent nuclei and ganglia. There are four light-modulated autonomic pathways: (1) parasympathetic (light-stimulated): PON→EW→CG →pupil constriction; (2) sympathetic (light-inhibited): SCN→PVN→IML→SCG→pupil dilation; (3) sympathetic (light-stimulated), SCN→DMH→LC→IML→SCG→pupil dilation; (4) sympathetic (light-inhibited): pretectum→PAG→sympathetic premotor nuclei (PVN, LC, DR)→IML→SCG→pupil dilation; CG—ciliary ganglion; DMH—dorsomedial hypothalamus; DRN—dorsal raphe nucleus; EWpg—Edinger-Westphal nucleus; IML—intermediolateral column; LC—locus coeruleus; oc—optic chiasm; PAG—periaqueductal gray autonomic ganglia; PON—pretectal olivary nucleus; PVN—paraventricular nucleus; SCG—superior cervical ganglion; SCN—suprachiasmatic nucleus.

Traditionally, the PLR is divided into afferent and efferent pathways based on clinical manifestations. As shown in Figure 2, after exposure to light, the retinal cells become activated and produce a signal, which is finally transmitted to the pretectal olivary nucleus (PON). This process is driven by the retinal rods and cones, as well as by the intrinsically photosensitive retinal ganglion cells (ipRGCs) [129,130]. The specific central projection of the afferent pathway is only known in mammals and birds in detail. Before 2002, the rods and cones were considered to be the only photoreceptors involved in the PLR [131]. In the subsequent 20 years, a non-rod, non-cone, and circadian-related photoreceptor have also been identified; these were eventually defined as ipRGCs in primates and rodents [132,133,134]. The effects of ipRGCs can be directly observed as they express melanopsin, which acts via the G-protein signaling cascades to mediate sustained pupil constriction [135]. Melanopsin gene knockout mice do not show intrinsic photosensitivity, suggesting diminished PLR [136]. This PLR response is also lost in mice lacking rods/cones and melanopsin [137]. Moreover, selective ablation of the ipRGCs causes the loss of PLR [138]. These findings highlight the central role of ipRGCs in non-image forming visual response.

The efferent pathway includes the projection from the PON to the EWpg and ends up at the sphincter pupillae muscle, from where the postganglionic fibers release acetylcholine (ACh), which acts on the muscarinic receptors on the sphincter muscles and leads directly to pupillary constriction [139]. This physiological process is observed to be related to changes in extracellular Ca^2+^ concentration and ryanodine receptor (RyR) channels in the iris sphincter muscle [126]. Pupillary dynamics depend on time, intensity of illumination, and the pupil shape in different species [128].

#### 4.1.2. Sympathetic Influences

It is generally accepted that light induces pupil contraction via the activation of the parasympathetic pathway, while the sympathetic system only has a tonic role [134]. The center of the sympathetic pathway is located at the locus coeruleus (LC), which contains both sympathetic neurons projecting to the SCG and parasympathetic neurons projecting to the EWn [140,141]. Generally, LC increases sympathetic activity via the activation of α1-adrenoceptors on preganglionic sympathetic neurons and reduces parasympathetic activity via the activation of α2-adrenoceptors on preganglionic parasympathetic neurons [142]. However, there is species difference in the function of the LC since the sympathetic system is also activated by the circadian system inputs. In diurnal species, the α2-adrenoceptors agonist mainly stimulates autoreceptors on sympathetic premotor neurons, causing miosis. While in nocturnal animals it stimulates postsynaptic α2-arenoceptors in the EWn, causing mydriasis [143].

Previous research has found the existence of neural connection from the pretectum to cervical sympathetic nerves [144]. Meanwhile, the PAG was suggested to function as an integrative relay nucleus in recent experiment and more likely to exert an inhibitory influence [145,146]. In diurnal species, a light-sensitive pathway (from retinal to the LC via the SCN and DMH) has also been identified, which mainly contributes to circadian regulation [147].

However, the physiological manipulations, such as the presentation of noxious or anxiety-provoking stimuli and extremes in ambient temperature, also affect the activity of LC, which lead to distinct patterns of change in arousal and autonomic function [148]. The pupil also dilates and contracts during cognitive and emotional processes [149,150,151,152]. The structures involved in this process may include the LC, colliculi, and prefrontal cortex [153,154,155]. However, the specific circuits mediating these responses remain unclear [156]. In brief, the sympathetic pathway may play a complementary role and may not contribute to the PLR dynamics [157].

### 4.2. Ocular Blood Flow

There are no blood vessels in the normal cornea, while the limbus contains abundant blood vessels that supply nutrients and oxygen. Similar to the cornea, the scleral stromal layer has no blood vessels, except those passing through. However, the sclera surface and optic nerve lamina cribrosa are rich in blood vessels and form a vascular network. These arteries are derived from the ophthalmic artery, which is the only artery supplying the eye. This blood vessel receives autonomic innervation and mainly supplies the uvea and retina. Hence, nearly all ocular circulation is modulated by autonomic nerves, except the retinal blood vessels [100].

The choroidal circulatory system is responsible for supplying the photoreceptors and the retinal pigment epithelium. Reduced blood flow can cause a rapid loss of photoreceptor cells [158]. Generally, autonomic innervation of the choroid includes parasympathetic pathways that dilate vessels and increase blood flow, sympathetic pathways that constrict vessels and decrease blood flow, and the local trigeminal system that transfers sensory input, or directly releases SP/CGRP in response to activating stimuli [29].

The SSN–PPG circuit mediates choroidal parasympathetic vasodilation, which seems to contribute to ChBF pressure regulation in case of low arterial blood pressure (ABP) [32,33]. In mammals, the PPG receives parasympathetic input from the SSN [35], and its preganglionic root, which contains NOS, VIP, and ChAT, directly projects to the choroid [159]. Many studies have discussed the role of VIP, NO, and ACh in mediating ChBF after stimulation of the facial nerve or SSN. Intravenous injection of VIP leads to increased intraocular pressure (IOP) and ChBF [160]. Different vasoactive substances affect the excitation frequencies of different nerves. In rabbits, the formation of NO (endothelial or neurogenic) is involved in uveal vasodilation caused by low-frequency facial nerve stimulation, while at high frequencies, other neurotransmitters also seem to be involved [161]. ChBF was significantly increased following facial nerve stimulation in monkeys, cats, and rabbits. Moreover, VIP was suggested as the peripheral molecule causing the vasodilation [162].

Based on immunohistochemical experiments in pigeons, the PPG seem to be composed of three to four main sub-ganglia connected to each other. Each main nerve contains 50–200 neurons, as well as several small ganglia. These neurons in birds release VIP and NO, and possibly Ach [163]. In mammals, the regulatory effect of PPG on ChBF appears to be similar as that in birds and can partially compensate for the decreased ABP. This may be a common ocular mechanism in warm-blooded vertebrates [164]. The avian choroid has a distinctive parasympathetic input of the CG and occupies a dominant position [29]. Using anatomical knowledge and electrical stimulation experiments, the central components from circuits in avian ChBF regulation were identified as follows: the retina–contralateral SCN that contains SP (+) neurons–medial EW (EWm), which controls ChBF via its ipsilateral projection to choroidal neurons of the CG [165]. Cantwell and Cassone indicated that SCN in avians can be divided into medial SCN (mSCN) and visual SCN (vSCN), while the latter is considered to participate in ChBF regulation [166,167]. If SCN was activated by retinal illumination of the contralateral eye, the choroidal volume appeared to increase (vasodilation) corresponding to a similar increase in systemic blood pressure [168]. This complex parasympathetic reflex response might be adaptive and is involved in maintaining the health of photoreceptor cells [169]. Cholinergic fibers of the CG are widely distributed in the choroid of avians. M2, M3, and M4 type receptors have been found in the retina, retinal pigment epithelium, choroid, and ciliary body [170]. After specifically suppressing these muscarinic receptors, M3 muscarinic receptors were observed to dominantly facilitate the EW-mediated increase in ChBF, with endothelial cell stimulation to release NO [171].

In both mammals and birds, the sympathetic nerves innervating the choroid are derived from noradrenergic nerve fibers of the cervical ganglia [29]. Stimulating the unilateral sympathetic nerve causes a large reduction in the ChBF [172,173]. After ICN transection, the choroids demonstrate increased vascularity and sympathetic denervation of the choroid and retinal defects [174,175]. These effects may be mediated by adrenoceptors. Previous research suggested that α-and β-adrenergic blocks can cause choroidal vasodilation and vasoconstriction, respectively, in rabbits [176]. This result is consistent with the observation that venous NPY treatment significantly reduces ChBF [177]. The trigeminal nerve branches that contain SP and CGRP innervate the choroid in mammals and birds [27]. Trigeminal nerve stimulation leads to the local release of vasodilators, SP, and CGRP [178]. Some researchers have recognized that the TG may be involved in the temperature-dependent regulation of ChBF. However, the specific underlying mechanisms need to be further investigated [29].

### 4.3. Intraocular Pressure Regulation

Aqueous humor (AH) is a transparent fluid found in the anterior and posterior chambers. It is mainly responsible for nutrient delivery and IOP regulation. It is produced by the ciliary epithelium and exits the eye through two independent outflow pathways, which involves the trabecular meshwork (TM) and unconventional pathway (uveoscleral). Recent evidence has suggested that another unconventional pathway (uveolymphatic route) has potential for maintaining IOP [179].

#### 4.3.1. Ciliary Body Blood Flow

The parasympathetic innervation of ciliary body blood flow is mediated by the postganglionic nerve fibers (VIP+) of the PPG [180,181]. Stimulation of the facial nerve and subsequent activation of PPG results in vasodilation and increased blood flow, and the contributing neurotransmitter is thought to be VIP [162]. Apart from ChAT and VIP release, NO may also participate in this process, especially at a low frequency of facial nerve stimulation [161,182]. The effect of NO has been proven since ciliary blood vessels constricted and aqueous production decreased after the inhibition of NO synthase [183].

Noradrenergic postganglionic neurons in the SCG provide sympathetic innervation. Unilateral sympathetic nerve stimulations (SNS) in rabbits have suggested frequency-dependent sympathetic vasoconstriction in the eyes [184]. The microstructure of the ciliary body in cats has revealed that the distribution of noradrenergic fibers is sparse in the TM and ciliary muscles, but denser in the subepithelial tissue [185]. Intravenous infusion of NPY causes a dose-dependent increase in total uveal vascular resistance with a decrease in blood flow in the ciliary body. This is consistent with the findings in rabbits whose α-adrenergic receptors were blocked, suggesting that NPY is also involved in sympathetic nerve-mediated vasoconstriction [177].

The trigeminal sensory nerves contain SP and CGRP, which are released as vasodilators in response to pressure or temperature stimuli. An intracameral injection of CGRP produced distinct vascular effects and increases IOP [186].

#### 4.3.2. AH Production

Aqueous humor production involves three mechanisms: active secretion, diffusion, and ultrafiltration. Of these, active secretion predominates and accounts for approximately 80–90%. Non-pigmented epithelial cells of the ciliary body are recognized as the sites for active AH secretion [187]. The secretion of AH is positively correlated with ciliary body blood flow until it reaches a critical level. Additionally, at that point, it becomes independent [188]. Based on this mechanism, the regulation of AH production partly depends on the indirect control of ciliary blood flow, in addition to the direct control of the cilia endothelium cells. As previously discussed, L-NAME (an inhibitor of NOS) causes ciliary vasoconstriction, thereby reducing aqueous humor production [183].

Neural modulation of AH production seems to be complicated and confusing. This conclusion is based on the existence of cholinergic and adrenergic receptors and second messenger systems in the ciliary epithelium, the diurnal variation in AH generation, and the various effects of drugs (Table 2) [183,189,190,191,192]. Generally, β-adrenergic agonists and VIP lead to an increase in AH, while α-2 adrenergic agonists, Ach muscarinic receptors and NPY reduce cAMP levels, thereby decreasing AH production [60]. Since postganglionic parasympathetic nerves release Ach at low frequency and VIP at high frequency, parasympathetic system may play contrary roles at different frequency.

#### 4.3.3. Conventional Outflow Pathway

**Figure 3:**
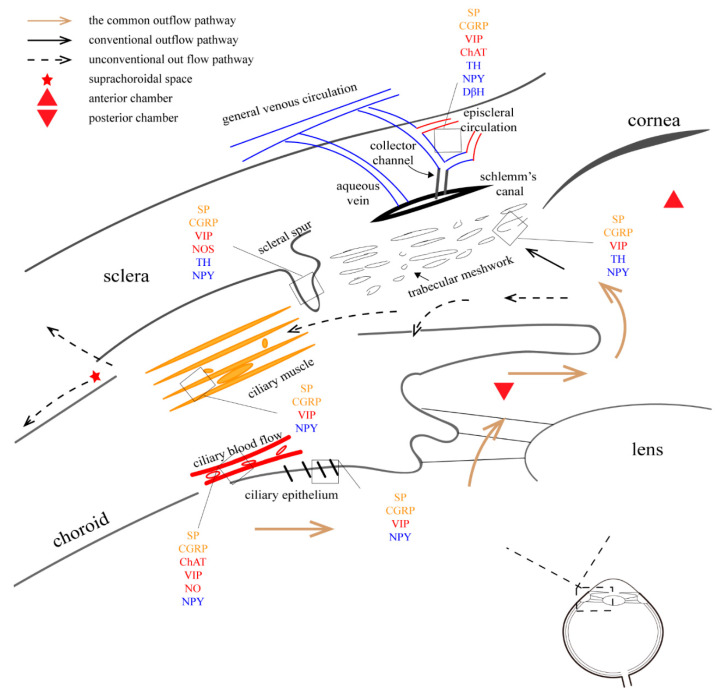
The aqueous humor outflow pathway. The red words represent parasympathetic innervation, the blue words represent sympathetic innervation, and the yellow words represent trigeminal innervation, respectively. There are two pathways of AH outflow: (1) conventional outflow pathway: non-pigmented epithelial cells→posterior chamber→anterior chamber→TM→Schlemm canal→collection tube, aqueous vein→episcleral vasculature→general venous circulation; (2) unconventional outflow pathway: non-pigmented epithelial cells→posterior chamber→anterior chamber→ciliary body clefts→interstitial spaces of the ciliary muscle→suprachoroidal space, vortex veins, and sclera. The uveolymphatic pathway remains controversial.

The AH is produced by the ciliary process. It enters the anterior chamber from the gap between the lens and the iris, passing through various areas of the TM and the inner wall of the Schlemm canal, before entering the superior scleral vein and returning to general venous circulation (Figure 3). The entire process may be an important component for sustaining IOP because most of AH flows along this path.

By contracting the ciliary muscles, muscarinic cholinergic agonist drugs mediate TM/SC relaxation and expansion, in which the iris does not participate [193]. In contrast, adrenaline and its second messenger cAMP are suggested to influence AH flow by directly acting on the endothelial cells of the trabecular meshwork–Schlemm’s canal inner wall [194,195]. Local application of 1% epinephrine led to a reduction in IOP with an increase in SC diameter, area, and TM width [196]. AH flow is also mediated by the iris and ciliary muscles. NOS in the endothelium mediates pressure-dependent drainage, which may form a negative feedback pathway [197]. A previous study has suggested that under electric stimulation of SCG in rats, the expression of DβH in the SC endothelium is increased, and the cross-sectional area and perimeter of the SC are reduced [198]. This may explain the elevated IOP [199]. Further investigations into the mechanisms of autonomic innervations are still needed.

#### 4.3.4. Unconventional Outflow Pathway

In the uveoscleral pathway, the AH flows into the ciliary body from the anterior chamber, enters the ciliary body clefts, passes through the suprachoroidal space, and leaves the eye through the vortex veins or into the sclera [200,201]. Johnson pointed out that ciliary body clefts may be key to this pathway because of their special anatomy [201]. A novel “uveolymphatic” outflow pathway was described in [202].

The amount of outflow via the unconventional route varies among different species, approximately ranging from 3% to 82% [201]. Thus, the outflow amount seems to depend on the development of the ciliary muscle [200]. Based on its anatomical characteristics, cholinergic drugs can cause ciliary muscle contraction, thereby significantly impeding uveoscleral outflow [203]. Adrenergic drugs also affect efflux through unconventional pathways, although the mechanism remains unclear. In monkeys, adrenaline doubles the uveoscleral flow [204].

#### 4.3.5. Episcleral Circulation

The episcleral vasculature contains numerous arteriovenous anastomoses and a muscle-rich venous network, which provide an anatomical basis for autonomic innervation [205,206]. Since AH leaves the eye through this structure, the specialized morphology allows the regulation of episcleral venous pressure (EVP) and IOP [207]. This may explain the increase in EVP after the electrical stimulation of SSN in rats [208].

Previous research on primate animals described numerous nerve endings staining for NAPDH diaphorase, TH, and relatively fewer NPY, VIP, and vesicular acetylcholine transporter-positive nerve terminals surrounded all the episcleral vessels; meanwhile, CGRP and SP-positive terminals were also observed [209]. Similar results in rabbits and rats have been published as well [206]. More recently, the immunoreactivity of episcleral vessels for synaptophysine, PGP 9.5, ChAT, DβH, VIP, CGRP, nNOS, SP, and galanin were proved in rats [210]. These neurotransmitters suggest the innervation of sympathetic and parasympathetic nervous system as well as sensory nerves.

### 4.4. Lens Accommodation

The accommodation reflex, also referred as near reflex, is the response for focusing on near objects [211]. The afferent limb of this reflex is through the optic nerve and efferent limb was considered to be the EWpg, EWcp, and the oculomotor [212]. The final effects of this reflex consists the convergence of both eyes and contraction of the ciliary muscle leading to the change of lens accommodation and pupillary constriction [213]. The ciliary muscle of mammals, and its homologues in fish and amphibia are contracted via cholinergic muscarinic mechanisms, while in birds and reptiles, acetylcholine acts via nicotinic receptors [60].

Recent research on primates suggested that the premotor neurons controlling the lens of unilateral eye are distributed in the bilateral midbrain. By retrograde tracing methods, some of the premotor neurons in the supraoculomotor area and central mesencephalic formation were doubly labeled, while others were labeled from either the ipsilateral or contralateral eye, which suggest the both monocular control and binocular control of lens accommodation [214].

Because of all the recent and past work that has been performed, lens accommodation to vergence angle and other aspects of eye movements are connected [214,215,216,217]. A cohort study showed that with lens accommodation, anterior chamber depth, and anterior chamber angle remained stable while the pupil diameter varied [218].

## 5. Clinical Application

Because of all the characteristics of anatomy and physiological effects discussed here, more and more studies have focused on the clinical applications of ANS agents. Apart from the drugs listed in the above tables, some other progress is described below.

Muscarinic receptor inhibitor (such as atropine) was proved to participate in the eye growth regulation and inhibit myopia induction in both mammalian and avian eyes [219,220]. Additionally, atropine is currently considered the most effective therapy for myopia control [221]. In addition, its topical application was demonstrated to stimulate subtypes of muscarinic receptor in scleral fibroblasts and inhibit scleral proliferation and matrix synthesis—the further mechanism remains unclear [222,223,224]. In the long term, this non-specific mAChR antagonist might cause premature presbyopia, cataract, and light damage in the retina [225].

Pilocarpine, a muscarinic receptor agonist, was observed to cause axial thickening of the lens and shallowing of the anterior chamber after instillation [226,227]. A multicentric retrospective study demonstrated that the topical use of pilocarpine significantly improved both uncorrected near visual acuity and uncorrected distance visual acuity, with spontaneously resolved side effects [228]. This result was consistent with another similar study and provide a new choice for those patients with presbyopia who do not wish to wear glasses [229].

As a specific agonist of the M3 muscarinic receptor, cevimeline has beneficial effects on dry eye symptoms in patients with Sjögren’s syndrome [230,231]. However, the question of how cevimeline improves dry eye and whether cevimeline increases cellular water transportation in the acinar and ductal cells of lacrimal glands still need further investigation.

The neuroprotective effects of NPY were confirmed by attenuating retinal neural apoptosis and maintain inner retinal vascular integrity. This neuropeptide may have potential therapy values in diabetic retinopathy [232].

## 6. Conclusions

This review highlights the multiple roles of the ANS in controlling the physiological processes of the eye. These roles include the adjustment of the pupils exposed to ambient light, ocular circulation in each tissue, and IOP regulation. Through these processes, the ANS contributes to regulating the accommodative status and sustaining intraocular homeostasis. Although differences exist in the precise wiring to the targets and innervation patterns among species, these autonomic nerves play an irreplaceable role in the eye.

Owing to the relationship between autonomic disorders and clinical problems, several commonly used drugs, such as cholinergic and adrenergic drugs, function through these autonomic pathways to correct disordered physiological processes. However, current drugs have their limitations. Understanding the role of the ANS will enhance the understanding of disease mechanisms that may subsequently lead to new drug therapies.

It has become increasingly apparent that these autonomic pathways are far more complex than that which has been explored to date. In the future, refining our understanding of the ocular ANS will provide new ideas for further investigations. Here, we list some urgent problems that need to be elucidated, as follows:

(1) Central control of physiological processes in the eye was hard to detect and clarify. However, the development of new techniques makes it possible to provide new insights into the central control of the eye.

(2) Detailed mechanisms of the AH outflow pathway. What we know about AH outflow pathway is pretty superficial. We still have a long way to go in understanding the normal physiology, or even the basic pathophysiology of the outflow dysfunction leading to POAG.

(3) Pathways of transformation from visual information to autonomic activity. The ipRGCs are activated by ambient lighting projected widely throughout the brain, subsequently mediating visual functions and circadian rhythms. Our mood, alertness, learning, regulation of body, and even visual perception are affected as well. However, the question of how this process works remains unclear and needs further identification.

## Figures and Tables

**Table 1:** Various peptide and transmitters in the cornea of mammals.

Compound.	Mechanism
Substance P	Epithelial renewal and wound repair
CGRP	Epithelial renewal and wound repair
Norepinephrine	Epithelial renewal and wound repair; stimulate proliferation and migration of corneal epithelial cells
Acetylcholine	Promote DNA synthesis in epithelial cells
VIP	Protect corneal endothelial cells from oxidative stress
Neurotensin	Increases keratocyte proliferation; decreases keratocycte apoptosis
Nerve growth factor	Sustain homeostasis and regeneration of epithelium and stroma

**Table 2:** Drugs and related mechanism.

Drug	Target	Mechanism
Cholinergic drug	Ciliary muscle	Increase in AH conventional outflow Decrease in AH unconventional outflow
Carbonic anhydrase inhibitor	Ciliary body; non-pigmented epithelial cells	Decrease in AH inflow
Epinephrine and analogue	Ciliary muscle; endothelial cells of the canal	Increase in AH conventional outflow Increase in unconventional outflow
β-Blocker	Ciliary body; non-pigmented epithelial cells	Decrease in AH inflow Increase in AH transscleral outflow
α2-Agonist	Ciliary body; non-pigmented epithelial cells	Decrease in AH inflow Increase in AH unconventional outflow
Prostaglandin and analogue	Ciliary muscle and extracellular matrix	Increase in AH conventional outflow Increase in AH unconventional outflow Increase in AH transscleral outflow Increase in AH uveolymphatic outflow

## Abbreviations

ABP	arterial blood pressure
AH	aqueous humor
ANS	autonomic nervous system
CG	ciliary ganglion
CGRP	calcitonin gene-related peptide
ChAT	choline acetyltransferase
ChBF	choroidal blood flow
CRA	central retinal artery
DMH	dorsomedial hypothalamus
DRN	dorsal raphe nucleus
EVP	episcleral venous pressure
EW	Edinger-Westphal
GABA	γ-aminobutyric acid
ICN	intrinsic choroidal neurons
IML	intermediolateral cell column
IOP	intraocular pressure
IR	immunoreactive
LC	locus coeruleus
LI	like immunoreactive
NOS	nitric oxide synthase
NPY	neuropeptide Y
PAG	periaqueduct grey
PLR	pupillary light reflex
PMR	photomechanical responses
PON	pretectal olivary nucleus
PPG	pterygopalatine ganglion
PVN	paraventricular nucleus
RyR	ryanodine receptor
SC	schlemm
SCG	superior cervical ganglion
SCN	suprachiasmatic nucleus
SEP	subepithelial plexus
SNS	sympathetic nerve stimulations
SP	substance P
SSN	superior salivatory nucleus
TG	trigeminal ganglion
TH	tyrosine hydroxylase
TM	trabecular meshwork
VIP	vasoactive intestinal polypeptide

## References

[1] Wang Y., Zekveld A., Naylor G., Ohlenforst B., Jansma E., Lorens A., Lunner T., Kramer S.E. (2016). Parasympathetic Nervous System Dysfunction, as Identified by Pupil Light Reflex, and Its Possible Connection to Hearing Impairment. PLoS ONE.

[2] Gibbins I. (2013). Functional organization of autonomic neural pathways. Organogenesis.

[3] Wehrwein E.A., Orer H.S., Barman S.M. (2016). Overview of the Anatomy, Physiology, and Pharmacology of the Autonomic Nervous System. Compr. Physiol..

[4] Yamaguchi K. (2017). Development of the human oculomotor nuclear complex: Centrally-projecting Edinger-Westphal nucleus. Neurosci. Lett..

[5] Vasconcelos L.A., Donaldson C., Sita L.V., Casatti C., Lotfi C.F., Wang L., Cadinouche M.A., Elias C.F., Lovejoy D.A., Bittencourt J. (2003). Urocortin in the central nervous system of a primate (*Cebus apella*): Sequencing, immunohistochemical, and hybridization histochemical characterization. J. Comp. Neurol..

[6] Horn A.K., Eberhorn A., Härtig W., Ardeleanu P., Messoudi A., Büttner-Ennever J.A. (2008). Perioculomotor cell groups in monkey and man defined by their histochemical and functional properties: Reappraisal of the Edinger-Westphal nucleus. J. Comp. Neurol..

[7] Reiner A., Erichsen J.T., Cabot J.B., Evinger C., Fitzerald M.E.C., Karten H.J. (1991). Neurotransmitter organization of the nucleus of Edinger-Westphal and its projection to the avian ciliary ganglion. Vis. Neurosci..

[8] Horn A.K., Schulze C., Radtke-Schuller S. (2009). The Edinger-Westphal Nucleus Represents Different Functional Cell Groups in Different Species. Ann. N. Y. Acad. Sci..

[9] Kozicz T., Bittencourt J., May P.J., Reiner A., Gamlin P., Palkovits M., Horn A., Toledo C.A., Ryabinin A.E. (2010). The Edinger-Westphal nucleus: A historical, structural, and functional perspective on a dichotomous terminology. J. Comp. Neurol..

[10] Ryabinin A., Tsivkovskaia N.O., Ryabinin S.A. (2005). Urocortin 1-containing neurons in the human Edinger-Westphal nucleus. Neuroscience.

[11] May P.J., Sun W., Wright N.F., Erichsen J.T. (2019). Pupillary light reflex circuits in the macaque monkey: The preganglionic Edinger-Westphal nucleus. Brain Struct. Funct..

[12] Warwick R. (1954). The ocular parasympathetic nerve supply and its mesencephalic sources. J. Anat..

[13] Erichsen J.T., May P.J. (2002). The pupillary and ciliary components of the cat Edinger-Westphal nucleus: A transsynaptic transport investigation. Vis. Neurosci..

[14] D’Antoni A.V. (2016). Gray’s Anatomy, the Anatomical Basis of Clinical Practice. Clinical Anatomy.

[15] Haładaj R. (2019). Anatomical variations of the ciliary ganglion with an emphasis on the location in the orbit. Anat. Sci. Int..

[16] Bhardwaj N., Joshi A. (2021). Neuroanatomy, Ciliary Ganglion. StatPearls.

[17] Kirch W., Neuhuber W., Tamm E.R. (1995). Immunohistochemical localization of neuropeptides in the human ciliary ganglion. Brain Res..

[18] May P.J., Warren S. (1993). Ultrastructure of the macaque ciliary ganglion. J. Neurocytol..

[19] Barnerssoi M., May P.J., Horn A.K.E. (2016). GABAergic innervation of the ciliary ganglion in macaque monkey—A light and electron microscopic study. J. Comp. Neurol..

[20] Kuwayama Y., Grimes P.A., Ponte B., Stone R.A. (1987). Autonomic neurons supplying the rat eye and the intraorbital distribution of vasoactive intestinal polypeptide (VIP)-like immunoreactivity. Exp. Eye Res..

[21] Grimes P.A., Koeberlein B., Tigges M., Stone R.A. (1998). Neuropeptide Y-like immunoreactivity localizes to preganglionic axon terminals in the rhesus monkey ciliary ganglion. Investig. Ophthalmol. Vis. Sci..

[22] Tsibul’Kin A.G., Kolesnikov L.L. (2004). Human and animal ciliary ganglion—History and modern conceptions. Morfologiia.

[23] Kaleczyc J., Juranek J., Całka J., Lakomy M. (2005). Immunohistochemical characterization of neurons in the porcine ciliary ganglion. Pol. J. Vet. Sci..

[24] Sun W., Erichsen J.T., May P.J. (1994). NADPH-diaphorase reactivity in ciliary ganglion neurons: A comparison of distributions in the pigeon, cat, and monkey. Vis. Neurosci..

[25] Kuchiiwa S., Kuchiiwa T., Suzuki T. (1989). Comparative anatomy of the accessory ciliary ganglion in mammals. Anat. Embryol..

[26] Park H.-Y.L., Jung S.H., Park S.-H., Park C.K. (2019). Detecting autonomic dysfunction in patients with glaucoma using dynamic pupillometry. Medicine.

[27] Stone R.A., Kuwayama Y., Laties A.M. (1987). Regulatory peptides in the eye. Experientia.

[28] Cuthbertson S., White J., Fitzgerald M.E., Shih Y.-F., Reiner A. (1996). Distribution within the choroid of cholinergic nerve fibers from the ciliary ganglion in pigeons. Vis. Res..

[29] Reiner A., Fitzgerald M.E., Del Mar N., Li C. (2018). Neural control of choroidal blood flow. Prog. Retin. Eye Res..

[30] Goadsby P.J. (2013). Autonomic nervous system control of the cerebral circulation. Handb. Clin. Neurol..

[31] Li C., Fitzgerald M.E., LeDoux M.S., Gong S., Ryan P., Del Mar N., Reiner A. (2010). Projections from the hypothalamic paraventricular nucleus and the nucleus of the solitary tract to prechoroidal neurons in the superior salivatory nucleus: Pathways controlling rodent choroidal blood flow. Brain Res..

[32] Li C., Fitzgerald M.E.C., Del Mar N., Cuthbertson-Coates S., LeDoux M.S., Gong S., Ryan J.P., Reiner A. (2015). The identification and neurochemical characterization of central neurons that target parasympathetic preganglionic neurons involved in the regulation of choroidal blood flow in the rat eye using pseudorabies virus, immunolabeling and conventional pathway tracing methods. Front. Neuroanat..

[33] Li C., Fitzgerald M.E., Del Mar N., Wang H., Haughey C., Honig M.G., Reiner A. (2021). Role of the superior salivatory nucleus in parasympathetic control of choroidal blood flow and in maintenance of retinal health. Exp. Eye Res..

[34] Lütjen-Drecoll E. (2006). Choroidal innervation in primate eyes. Exp. Eye Res..

[35] Tusscher M.P.T., Klooster J., Baljet B., Van der Werf F., Vrensen G.F. (1990). Pre- and post-ganglionic nerve fibres of the pterygopalatine ganglion and their allocation to the eyeball of rats. Brain Res..

[36] Beckers H., Klooster J., Vrensen G., Lamers W. (1993). Facial Parasympathetic Innervation of the Rat Choroid, Lacrimal Glands and Ciliary Ganglion. Ophthalmic Res..

[37] Botelho S.Y., Hisada M., Fuenmayor N. (1966). Functional Innervation of the Lacrimal Gland in the Cat. Arch. Ophthalmol..

[38] Ruskell G.L. (1971). The distribution of autonomic post-ganglionic nerve fibres to the lacrimal gland in monkeys. J. Anat..

[39] Ackerknecht E.H. (1974). The history of the discovery of the vegatative (autonomic) nervous system. Med. Hist..

[40] Lee J.Y., Lee J.H., Song J.S., Song M.J., Hwang S.-J., Yoon R.G., Jang S.W., Park J.E., Heo Y.J., Choi Y.J. (2016). Superior Cervical Sympathetic Ganglion: Normal Imaging Appearance on 3T-MRI. Korean J. Radiol..

[41] Sonne J., Lopez-Ojeda W. (2021). Neuroanatomy, Cranial Nerve. StatPearls.

[42] Ebbesson S.O.E. (1968). Quantitative studies of superior cervical sympathetic ganglia in a variety of primates including man. I. The ratio of preganglionic fibers to ganglionic neurons. J. Morphol..

[43] Brooks-Fournier R., Coggeshall R.E. (1981). The ratio of preganglionic axons to postganglionic cells in the sympathetic nervous system of the rat. J. Comp. Neurol..

[44] Purves D., Rubin E., Snider W., Lichtman J. (1986). Relation of animal size to convergence, divergence, and neuronal number in peripheral sympathetic pathways. J. Neurosci..

[45] Tusscher M.T., Klooster J., van der Want J., Lamers W., Vrensen G. (1989). The allocation of nerve fibres to the anterior eye segment and peripheral ganglia of rats. II. The sympathetic innervation. Brain Res..

[46] Huff T., Daly D.T. (2021). Neuroanatomy, Cranial Nerve 5 (Trigeminal). StatPearls.

[47] Terenghi G., Polak J.M., Ghatei M.A., Mulderry P.K., Butler J.M., Unger W.G., Bloom S.R. (1985). Distribution and origin of calcitonin gene-related peptide (CGRP) immunoreactivity in the sensory innervation of the mammalian eye. J. Comp. Neurol..

[48] Lee Y., Kawai Y., Shiosaka S., Takami K., Kiyama H., Hillyard C., Girgis S., MacIntyre I., Emson P., Tohyama M. (1985). Coexistence of calcitonin gene-related peptide and substance P-like peptide in single cells of the trigeminal ganglion of the rat: Immunohistochemical analysis. Brain Res..

[49] Yang A.Y., Chow J., Liu J. (2018). Corneal Innervation and Sensation: The Eye and Beyond. Yale J. Biol. Med..

[50] Marfurt C.F., Kingsley R.E., Echtenkamp S.E. (1989). Sensory and sympathetic innervation of the mammalian cornea. A retrograde tracing study. Investig. Ophthalmol. Vis. Sci..

[51] Müller L.J., Marfurt C.F., Kruse F., Tervo T.M. (2003). Corneal nerves: Structure, contents and function. Exp. Eye Res..

[52] Medeiros C.S., Santhiago M.R. (2020). Corneal nerves anatomy, function, injury and regeneration. Exp. Eye Res..

[53] Zander E., Weddell G. (1951). Observations on the innervation of the cornea. J. Anat..

[54] Al-Aqaba M.A., Fares U., Suleman H., Lowe J., Dua H.S. (2009). Architecture and distribution of human corneal nerves. Br. J. Ophthalmol..

[55] Müller L.J., Pels L., Vrensen G.F. (1996). Ultrastructural organization of human corneal nerves. Investig. Ophthalmol. Vis. Sci..

[56] Stern M.E., Beuerman R.W., Fox R.I., Gao J., Mircheff A.K., Pflugfelder S.C. (1998). The Pathology of Dry Eye. Cornea.

[57] Ghiasi Z., Gray T., Tran P., Dubielzig R., Murphy C., McCartney D.L., Reid T.W. (2018). The Effect of Topical Substance-P Plus Insulin-like Growth Factor-1 (IGF-1) on Epithelial Healing After Photorefractive Keratectomy in Rabbits. Transl. Vis. Sci. Technol..

[58] Suvas S. (2017). Role of Substance P Neuropeptide in Inflammation, Wound Healing, and Tissue Homeostasis. J. Immunol..

[59] Nakamura M., Nishida T., Ofuji K., Reid T.W., Mannis M.J., Murphy C.J. (1997). Synergistic Effect of Substance P with Epidermal Growth Factor on Epithelial Migration in Rabbit Cornea. Exp. Eye Res..

[60] Neuhuber W., Schrödl F. (2011). Autonomic control of the eye and the iris. Auton. Neurosci..

[61] Kawasaki A. (1999). Physiology, assessment, and disorders of the pupil. Curr. Opin. Ophthalmol..

[62] Stone R., Laties A., Brecha N. (1982). Substance P-like immunoreactive nerves in the anterior segment of the rabbit, cat and monkey eye. Neuroscience.

[63] Fujiwara M., Hayashi H., Muramatsu I., Ueda N. (1984). Supersensitivity of the rabbit iris sphincter muscle induced by trigeminal denervation: The role of substance P. J. Physiol..

[64] Bremner F. (2009). Pupil evaluation as a test for autonomic disorders. Clin. Auton. Res..

[65] Hardie R.C., Franze K. (2012). Photomechanical Responses in Drosophila Photoreceptors. Science.

[66] Barr L. (1989). Photomechanical coupling in the vertebrate sphincter pupillae. Crit. Rev. Neurobiol..

[67] Rubin L.J., Nolte J.F. (1984). Modulation of the response of a photosensitive muscle by β-adrenergic regulation of cyclic AMP levels. Nature.

[68] Tamm E.R. (2009). The trabecular meshwork outflow pathways: Structural and functional aspects. Exp. Eye Res..

[69] Ruskell G.L. (1994). Trigeminal innervation of the scleral spur in cynomolgus monkeys. J. Anat..

[70] Tamm E.R., Koch T.A., Mayer B., Stefani F.H., Lütjen-Drecoll E. (1995). Innervation of myofibroblast-like scleral spur cells in human monkey eyes. Investig. Ophthalmol. Vis. Sci..

[71] Stone R.A. (1986). Neuropeptide Y and the innervation of the human eye. Exp. Eye Res..

[72] Stone R.A., Tervo T., Tervo K., Tarkkanen A. (1986). Vasoactive intestinal polypeptide-like immunoreactive nerves to the human eye. Acta Ophthalmol..

[73] Laties A.M., Stone R.A., Brecha N.C. (1981). Substance P-like immunoreactive nerve fibers in the trabecular meshwork. Investig. Ophthalmol. Vis. Sci..

[74] Stone R.A., McGlinn A.M. (1988). Calcitonin gene-related peptide immunoreactive nerves in human and rhesus monkey eyes. Investig. Ophthalmol. Vis. Sci..

[75] Selbach J.M., Gottanka J., Wittmann M., Lütjen-Drecoll E. (2000). Efferent and afferent innervation of primate trabecular meshwork and scleral spur. Investig. Ophthalmol. Vis. Sci..

[76] Yang F., Zhu X., Liu X., Ma L., Zhang Z., Pei L., Wang H., Xu F., Liu H. (2020). Anatomical evidence for the efferent pathway from the hypothalamus to autonomic innervation in the anterior chamber structures of eyes. Exp. Eye Res..

[77] Selbach J.M., Buschnack S.H., Steuhl K.-P., Kremmer S., Muth-Selbach U. (2005). Substance P and opioid peptidergic innervation of the anterior eye segment of the rat: An immunohistochemical study. J. Anat..

[78] May C.A., Skorski L.M., Lutjen-Drecoll E. (2005). Innervation of the porcine ciliary muscle and outflow region. J. Anat..

[79] Nomura T., Smelser G.K. (1974). The identification of adrenergic and cholinergic nerve endings in the trabecular meshwork. Investig. Ophthalmol..

[80] Jampel H.D., Lynch M.G., Brown R.H., Kuhar M.J., De Souza E.B. (1987). Beta-adrenergic receptors in human trabecular meshwork. Identification and autoradiographic localization. Investig. Ophthalmol. Vis. Sci..

[81] Wax M.B., Molinoff P.B., Alvarado J., Polansky J. (1989). Characterization of beta-adrenergic receptors in cultured human trabecular cells and in human trabecular meshwork. Investig. Ophthalmol. Vis. Sci..

[82] Dartt D.A. (2009). Neural regulation of lacrimal gland secretory processes: Relevance in dry eye diseases. Prog. Retin. Eye Res..

[83] Acosta M.C., Belmonte C., Gallar J. (2001). Sensory experiences in humans and single-unit activity in cats evoked by polymodal stimulation of the cornea. J. Physiol..

[84] Gallar J., Pozo M.A., Tuckett R.P., Belmonte C. (1993). Response of sensory units with unmyelinated fibres to mechanical, thermal and chemical stimulation of the cat’s cornea. J. Physiol..

[85] Acosta M.C., Peral A., Luna C., Pintor J., Belmonte C., Gallar J. (2004). Tear Secretion Induced by Selective Stimulation of Corneal and Conjunctival Sensory Nerve Fibers. Investig. Opthalmol. Vis. Sci..

[86] Botelho S., Martinez E., Pholpramool C., Prooyen H., Janssen J., De Palau A. (1976). Modification of stimulated lacrimal gland flow by sympathetic nerve impulses in rabbit. Am. J. Physiol. Content.

[87] Adeghate E., Singh J., Howarth F.C., Burrows S. (1997). Control of porcine lacrimal gland secretion by non-cholinergic, non-adrenergic nerves: Effects of electrical field stimulation, VIP and NPY. Brain Res..

[88] Ding C., Walcott B., Keyser K.T. (2003). Sympathetic Neural Control of the Mouse Lacrimal Gland. Investig. Opthalmol. Vis. Sci..

[89] Meneray M.A., Bennett D.J., Nguyen D.H., Beuerman R.W. (1998). Effect of Sensory Denervation on the Structure and Physiologic Responsiveness of Rabbit Lacrimal Gland. Cornea.

[90] Ruskell G.L. (1969). Changes in nerve terminals and acini of the lacrimal gland and changes in secretion induced by autonomic denervation. Z. Für Zellforsch. Und Mikrosk. Anat..

[91] Tangkrisanavinont V. (1984). Stimulation of lacrimal secretion by sympathetic nerve impulses in the rabbit. Life Sci..

[92] Jin K., Imada T., Hisamura R., Ito M., Toriumi H., Tanaka K., Nakamura S., Tsubota K. (2020). Identification of Lacrimal Gland Postganglionic Innervation and Its Regulation of Tear Secretion. Am. J. Pathol..

[93] Nakamura M., Tada Y., Akaishi T., Nakata K. (1997). M3 muscarinic receptor mediates regulation of protein secretion in rabbit lacrimal gland. Curr. Eye Res..

[94] Mauduit P., Jammes H., Rossignol B. (1993). M3 muscarinic acetylcholine receptor coupling to PLC in rat exorbital lacrimal acinar cells. Am. J. Physiol. Physiol..

[95] Toshida H., Nguyen A.H., Beuerman R.W., Murakami A. (2007). Evaluation of Novel Dry Eye Model: Preganglionic Parasympathetic Denervation in Rabbit. Investig. Opthalmol. Vis. Sci..

[96] Alm A., Bill A. (1973). Ocular and optic nerve blood flow at normal and increased intraocular pressures in monkeys (*Macaca irus*): A study with radioactively labelled microspheres including flow determinations in brain and some other tissues. Exp. Eye Res..

[97] Törnquist P., Alm A. (1979). Retinal and choroidal contribution to retinal metabolism in vivo. A study in pigs. Acta Physiol. Scand..

[98] Hogan M.J., Feeney L. (1963). The ultrastructure of the retinal blood vessels: I. The large vessels. J. Ultrastruct. Res..

[99] Laties A.M. (1967). Central Retinal Artery Innervation. Arch. Ophthalmol..

[100] Delaey C., Van De Voorde J. (2000). Regulatory Mechanisms in the Retinal and Choroidal Circulation. Ophthalmic Res..

[101] Ferrari-DiLeo G., Davis E.B., Anderson D.R. (1989). Biochemical evidence for cholinergic activity in retinal blood vessels. Investig. Ophthalmol. Vis. Sci..

[102] Bergua A., Kapsreiter M., Neuhuber W.L., Reitsamer H.A., Schrödl F. (2013). Innervation pattern of the preocular human central retinal artery. Exp. Eye Res..

[103] Bergua A., Schrödl F., Neuhuber W.L. (2003). Vasoactive intestinal and calcitonin gene-related peptides, tyrosine hydroxylase and nitrergic markers in the innervation of the rat central retinal artery. Exp. Eye Res..

[104] Ye X.D., Laties A.M., Stone R.A. (1990). Peptidergic innervation of the retinal vasculature and optic nerve head. Investig. Ophthalmol. Vis. Sci..

[105] Kumagai N., Yuda K., Kadota T., Goris R.C., Kishida R. (1988). Substance P-like immunoreactivity in the central retinal artery of the rabbit. Exp. Eye Res..

[106] Laties A.M., Jacobowitz D. (1966). A comparative study of the autonomic innervation of the eye in monkey, cat, and rabbit. Anat. Rec. Adv. Integr. Anat. Evol. Biol..

[107] Toda N., Toda M., Ayajiki K., Okamura T. (1996). Monkey central retinal artery is innervated by nitroxidergic vasodilator nerves. Investig. Ophthalmol. Vis. Sci..

[108] Toda N., Ayajiki K., Yoshida K., Kimura H., Okamura T. (1993). Impairment by damage of the pterygopalatine ganglion of nitroxidergic vasodilator nerve function in canine cerebral and retinal arteries. Circ. Res..

[109] Smith C.P., Sharma S., Steinle J. (2007). Age-related changes in sympathetic neurotransmission in rat retina and choroid. Exp. Eye Res..

[110] Steinle J.J., Lindsay N.L., Lashbrook B.L. (2005). Cervical sympathectomy causes photoreceptor-specific cell death in the rat retina. Auton. Neurosci..

[111] May C.A., Neuhuber W., Lütjen-Drecoll E. (2004). Immunohistochemical Classification and Functional Morphology of Human Choroidal Ganglion Cells. Investig. Opthalmol. Vis. Sci..

[112] Schrödl F., Brehmer A., Neuhuber W.L. (2000). Intrinsic choroidal neurons in the duck eye express galanin. J. Comp. Neurol..

[113] De Hoz R., Salazar J.J., Ramírez A.I., Rojas B., Triviño A., Ramírez J.M. (2006). Estudio comparativo de la inervación coroidea en el hombre y en el conejo (oryctolagus cuniculus). Arch. Soc. Esp. Oftalmol..

[114] Schroedl F., De Stefano M.E., Reese S., Brehmer A., Neuhuber W.L. (2004). Comparative anatomy of nitrergic intrinsic choroidal neurons (ICN) in various avian species. Exp. Eye Res..

[115] Terenghi G., Polak J.M., Probert L., McGregor G.P., Ferri G.L., Blank M.A., Butler J.M., Unger W.G., Zhang A.-Q., Cole D.F. (1982). Mapping, quantitative distribution and origin of substance P- and VIP-containing nerves in the Uvea of guinea pig eye. Histochemistry.

[116] Nakanome Y., Karita K., Izumi H., Tamai M. (1995). Two types of vasodilatation in cat choroid elicited by electrical stimulation of the short ciliary nerve. Exp. Eye Res..

[117] Gherezghiher T., Hey J.A., Koss M.C. (1990). Parasympathetic nervous control of intraocular pressure. Exp. Eye Res..

[118] Stjernschantz J., Bill A. (1979). Effect of intracranial stimulation of the oculomotor nerve on ocular blood flow in the monkey, cat, and rabbit. Investig. Ophthalmol. Vis. Sci..

[119] Triviño A., De Hoz R., Rojas B., Salazar J.J., Ramirez A.I., Ramirez J.M. (2005). NPY and TH innervation in human choroidal whole-mounts. Histol. Histopathol..

[120] Klooster J., Beckers H., Tusscher T., Vrensen G., Van Der Want J., Lamers W. (1996). Sympathetic Innervation of the Rat Choroid: An Autoradiographic Tracing and Immunohistochemical Study. Ophthalmic Res..

[121] Schrödl F., Tines R., Brehmer A., Neuhuber W.L. (2001). Intrinsic choroidal neurons in the duck eye receive sympathetic input: Anatomical evidence for adrenergic modulation of nitrergic functions in the choroid. Cell Tissue Res..

[122] May C.A. (2013). Chronologic versus Biologic Aging of the Human Choroid. Sci. World J..

[123] Jablonski M.M., Iannaccone A., Reynolds D.H., Gallaher P., Allen S., Wang X., Reiner A. (2007). Age-Related Decline in VIP-Positive Parasympathetic Nerve Fibers in the Human Submacular Choroid. Investig. Opthalmol. Vis. Sci..

[124] Nuzzi R., Finazzo C., Grignolo F.M. (1996). Changes in adrenergic innervation of the choroid during aging. J. Fr. Dophtalmol..

[125] Hoz A.D., Ramírez A.I., Salazar J.J., Rojas B., Ramírez J.M., Triviño A. (2008). Substance P and calcitonin gene-related peptide intrinsic choroidal neurons in human choroidal whole-mounts. Histol. Histopathol..

[126] Sghari S., Davies W.I.L., Gunhaga L. (2020). Elucidation of Cellular Mechanisms That Regulate the Sustained Contraction and Relaxation of the Mammalian Iris. Investig. Opthalmol. Vis. Sci..

[127] Lai J.S.M., Tham C.C.Y., Lam D.S.C. (2001). Comparative study of intraoperative mitomycin C and beta irradiation in pterygium surgery. Br. J. Ophthalmol..

[128] Douglas R.H. (2018). The pupillary light responses of animals; a review of their distribution, dynamics, mechanisms and functions. Prog. Retin. Eye Res..

[129] Zele A.J., Adhikari P., Cao D., Feigl B. (2019). Melanopsin and Cone Photoreceptor Inputs to the Afferent Pupil Light Response. Front. Neurol..

[130] Hayter E.A., Brown T.M. (2018). Additive contributions of melanopsin and both cone types provide broadband sensitivity to mouse pupil control. BMC Biol..

[131] Lucas R.J. (2013). Mammalian Inner Retinal Photoreception. Curr. Biol..

[132] Berson D.M., Dunn F.A., Takao M. (2002). Phototransduction by Retinal Ganglion Cells That Set the Circadian Clock. Science.

[133] Hattar S., Liao H.-W., Takao M., Berson D.M., Yau K.-W. (2002). Melanopsin-containing retinal ganglion cells: Architecture, projections, and intrinsic photosensitivity. Science.

[134] McDougal D.H., Gamlin P.D. (2014). Autonomic Control of the Eye. Compr. Physiol..

[135] Gamlin P.D., McDougal D.H., Pokorny J., Smith V.C., Yau K.-W., Dacey D.M. (2007). Human and macaque pupil responses driven by melanopsin-containing retinal ganglion cells. Vis. Res..

[136] Lucas R.J., Hattar S., Takao M., Berson D.M., Foster R.G., Yau K.-W. (2003). Diminished Pupillary Light Reflex at High Irradiances in Melanopsin-Knockout Mice. Science.

[137] Hattar S., Lucas R.J., Mrosovsky N., Thompson S., Douglas R.H., Hankins M.W., Lem J., Biel M., Hofmann F., Foster R.G. (2003). Melanopsin and rod–cone photoreceptive systems account for all major accessory visual functions in mice. Nature.

[138] Hatori M., Le H., Vollmers C., Keding S.R., Tanaka N., Schmedt C., Jegla T., Panda S. (2008). Inducible Ablation of Melanopsin-Expressing Retinal Ganglion Cells Reveals Their Central Role in Non-Image Forming Visual Responses. PLoS ONE.

[139] Bouffard M.A. (2019). The Pupil. Contin. Lifelong Learn. Neurol..

[140] Breen L.A., Burde R.M., Loewy A.D. (1983). Brainstem connections to the Edinger-Westphal nucleus of the cat: A retrograde tracer study. Brain Res..

[141] Jones B.E., Yang T.-Z. (1985). The efferent projections from the reticular formation and the locus coeruleus studied by anterograde and retrograde axonal transport in the rat. J. Comp. Neurol..

[142] Lewis D., Coote J. (1990). Excitation and inhibition of rat sympathetic preganglionic neurones by catecholamines. Brain Res..

[143] Szabadi E. (2018). Functional Organization of the Sympathetic Pathways Controlling the Pupil: Light-Inhibited and Light-Stimulated Pathways. Front. Neurol..

[144] Okada H., Nakano O., Okamoto K., Nakayama K., Nisida I. (1960). The central path of the light reflex via the sympathetic nerve in the cat. Jpn. J. Physiol..

[145] Dampney R.A., Furlong T., Horiuchi J., Iigaya K. (2013). Role of dorsolateral periaqueductal grey in the coordinated regulation of cardiovascular and respiratory function. Auton. Neurosci..

[146] Bajic D., Van Bockstaele E.J., Proudfit H.K. (2012). Ultrastructural analysis of rat ventrolateral periaqueductal gray projections to the A5 cell group. Neuroscience.

[147] González M.M., Aston-Jones G. (2006). Circadian regulation of arousal: Role of the noradrenergic locus coeruleus system and light exposure. Sleep.

[148] Samuels E., Szabadi E.R.S.A.E. (2008). Functional Neuroanatomy of the Noradrenergic Locus Coeruleus: Its Roles in the Regulation of Arousal and Autonomic Function Part II: Physiological and Pharmacological Manipulations and Pathological Alterations of Locus Coeruleus Activity in Humans. Curr. Neuropharmacol..

[149] Vanderhasselt M.-A., De Raedt R., Nasso S., Puttevils L., Mueller S. (2018). Don’t judge me: Psychophysiological evidence of gender differences to social evaluative feedback. Biol. Psychol..

[150] Lichtenstein-Vidne L., Gabay S., Cohen N., Henik A. (2016). Lateralisation of emotions: Evidence from pupil size measurement. Cogn. Emot..

[151] Brocher A., Graf T. (2017). Decision-related factors in pupil old/new effects: Attention, response execution, and false memory. Neuropsychologia.

[152] Gomes C.A., Montaldi D., Mayes A. (2015). The pupil as an indicator of unconscious memory: Introducing the pupil priming effect. Psychophysiology.

[153] Ebitz R.B., Moore T. (2017). Selective Modulation of the Pupil Light Reflex by Microstimulation of Prefrontal Cortex. J. Neurosci..

[154] Joshi S., Li Y., Kalwani R.M., Gold J.I. (2015). Relationships between Pupil Diameter and Neuronal Activity in the Locus Coeruleus, Colliculi, and Cingulate Cortex. Neuron.

[155] Wang C.-A.J., Boehnke S., White B.J., Munoz D.P. (2012). Microstimulation of the Monkey Superior Colliculus Induces Pupil Dilation Without Evoking Saccades. J. Neurosci..

[156] Peinkhofer C., Knudsen G.M., Moretti R., Kondziella D. (2019). Cortical modulation of pupillary function: Systematic review. PeerJ.

[157] Clarke R.J., Zhang H., Gamlin P.D.R. (2003). Characteristics of the Pupillary Light Reflex in the Alert Rhesus Monkey. J. Neurophysiol..

[158] Gaudric A., Coscas G., Bird A. (1982). Choroidal Ischemia. Am. J. Ophthalmol..

[159] Cuthbertson S., LeDoux M.S., Jones S., Jones J., Zhou Q., Gong S., Ryan P., Reiner A. (2003). Localization of preganglionic neurons that innervate choroidal neurons of pterygopalatine ganglion. Investig. Opthalmol. Vis. Sci..

[160] Nilsson S.F.E., Bill A. (1984). Vasoactive intestinal polypeptide (VIP): Effects in the eye and on regional blood flows. Acta Physiol. Scand..

[161] Nilsson S.F. (1996). Nitric oxide as a mediator of parasympathetic vasodilation in ocular and extraocular tissues in the rabbit. Investig. Ophthalmol. Vis. Sci..

[162] Nilsson S.F., Linder J., Bill A. (1985). Characteristics of uveal vasodilation produced by facial nerve stimulation in monkeys, cats and rabbits. Exp. Eye Res..

[163] Cuthbertson S., Jackson B., Toledo C., Fitzgerald M.E.C., Shih Y.F., Zagvazdin Y., Reiner A. (1997). Innervation of orbital and choroidal blood vessels by the pterygopalatine ganglion in pigeons. J. Comp. Neurol..

[164] Reiner A., Zagvazdin Y., Fitzgerald M.E. (2003). Choroidal blood flow in pigeons compensates for decreases in arterial blood pressure. Exp. Eye Res..

[165] Gamlin P., Reiner A., Karten H.J. (1982). Substance P-containing neurons of the avian suprachiasmatic nucleus project directly to the nucleus of Edinger-Westphal. Proc. Natl. Acad. Sci. USA.

[166] Cantwell E.L., Cassone V.M. (2006). Chicken suprachiasmatic nuclei: I. Efferent and afferent connections. J. Comp. Neurol..

[167] Cantwell E.L., Cassone V.M. (2006). Chicken suprachiasmatic nuclei: II. Autoradiographic and immunohistochemical analysis. J. Comp. Neurol..

[168] Fitzgerald M.E.C., Gamlin P.D.R., Zagvazdin Y., Reiner A. (1996). Central neural circuits for the light-mediated reflexive control of choroidal blood flow in the pigeon eye: A laser Doppler study. Vis. Neurosci..

[169] Reiner A., Wong T., Nazor C., DEL Mar N., Fitzgerald M. (2016). Type-specific photoreceptor loss in pigeons after disruption of parasympathetic control of choroidal blood flow by the medial subdivision of the nucleus of Edinger-Westphal. Vis. Neurosci..

[170] Fischer A.J., McKinnon L.A., Nathanson N.M., Stell W.K. (1998). Identification and localization of muscarinic acetylcholine receptors in the ocular tissues of the chick. J. Comp. Neurol..

[171] Zagvazdin Y., Fitzgeraldab M.E., Reiner A. (2000). Role of Muscarinic Cholinergic Transmission in Edinger-Westphal Nucleus-induced Choroidal Vasodilation in Pigeon. Exp. Eye Res..

[172] Alm A., Bill A. (1973). The Effect of Stimulation of the Cervical Sympathetic Chain on Retinal Oxygen Tension and on Uveal, Retinal and Cerebral Blood Flow in Cats. Acta Physiol. Scand..

[173] Alm A. (1977). The effect of sympathetic stimulation on blood flow through the uvea, retina and optic nerve in monkeys (*Macaca irus*). Exp. Eye Res..

[174] Li C., Fitzgerald M.E.C., Del Mar N., Haughey C., Reiner A. (2018). Defective Choroidal Blood Flow Baroregulation and Retinal Dysfunction and Pathology Following Sympathetic Denervation of Choroid. Investig. Opthalmol. Vis. Sci..

[175] Martinez-Camarillo J.-C., Spee C.K., Chen M., Rodriguez A., Nimmagadda K., Trujillo-Sánchez G.P., Hinton D.R., Giarola A., Pikov V., Sridhar A. (2019). Sympathetic Effects of Internal Carotid Nerve Manipulation on Choroidal Vascularity and Related Measures. Investig. Opthalmol. Vis. Sci..

[176] Kiel J.W., Lovell M.O. (1996). Adrenergic modulation of choroidal blood flow in the rabbit. Investig. Ophthalmol. Vis. Sci..

[177] Nilsson S.F.E. (1991). Neuropeptide Y (NPY): A vasoconstrictor in the eye, brain and other tissues in the rabbit. Acta Physiol. Scand..

[178] Stjernschantz J., Geijer C., Bill A. (1979). Electrical stimulation of the fifth cranial nerve in rabbits: Effects on ocular blood flow, extravascular albumin content and intraocular pressure. Exp. Eye Res..

[179] Costagliola C., Dell’Omo R., Agnifili L., Bartollino S., Fea A.M., Uva M.G., Zeppa L., Mastropasqua L. (2019). How many aqueous humor outflow pathways are there?. Surv. Ophthalmol..

[180] Ruskell G. (1970). An ocular parasympathetic nerve pathway of facial nerve origin and its influence on intraocular pressure. Exp. Eye Res..

[181] Uusitalo H., Lehtosalo J.I., Palkama A. (1985). Vasoactive Intestinal Polypeptide(VIP)-Immunoreactive Nerve Fibers in the Anterior Uvea of the Guinea Pig. Ophthalmic Res..

[182] Nilsson S.F. (2000). The Significance of Nitric Oxide for Parasympathetic Vasodilation in the Eye and other Orbital Tissues in the Cat. Exp. Eye Res..

[183] Kiel J.W., Reitsamerab H.A., Walker J.S., Kiel F.W. (2001). Effects of Nitric Oxide Synthase Inhibition on Ciliary Blood Flow, Aqueous Production and Intraocular Pressure. Exp. Eye Res..

[184] Granstam E., Nilsson S.F. (1990). Non-adrenergic sympathetic vasoconstriction in the eye and some other facial tissues in the rabbit. Eur. J. Pharmacol..

[185] Akagi Y., Ibata Y., Sano Y. (1976). The sympathetic innervation of the ciliary body and trabecular meshwork of the cat. Cell Tissue Res..

[186] Oksala O., Stjernschantz J. (1988). Effects of calcitonin gene-related peptide in the eye. A study in rabbits and cats. Investig. Ophthalmol. Vis. Sci..

[187] Goel M., Pacciani R.G., Lee R.K., Battacharya S.K. (2010). Aqueous Humor Dynamics: A Review. Open Ophthalmol. J..

[188] Kiel J., Hollingsworth M., Rao R., Chen M., Reitsamer H. (2011). Ciliary blood flow and aqueous humor production. Prog. Retin. Eye Res..

[189] Crosson C.K., Heath A.R., Devries G.W., Potter D.E. (1992). Pharmacological evidence for heterogeneity of ocular $aL2adrenoceptors. Curr. Eye Res..

[190] Wax M.B., Molinoff P.B. (1987). Distribution and properties of beta-adrenergic receptors in human iris-ciliary body. Investig. Ophthalmol. Vis. Sci..

[191] Polansky J.R., Zlock D., Brasier A., Bloom E. (1985). Adrenergic and cholinergic receptors in isolated non-pigmented ciliary epithelial cells. Curr. Eye Res..

[192] Ogidigben M., Chu T.C., Potter D.E. (1994). Ocular actions of moxonidine: A possible role for imidazoline receptors. J. Pharmacol. Exp. Ther..

[193] Kaufman P.L. (1979). Aqueous humor dynamics following total iridectomy in the cynomolgus monkey. Investig. Ophthalmol. Vis. Sci..

[194] Kaufman P.L., Bárány E.H. (1981). Adrenergic drug effects on aqueous outflow facility following ciliary muscle retrodisplacement in the cynomolgus monkey. Investig. Ophthalmol. Vis. Sci..

[195] Kaufman P.L. (1986). Total iridectomy does not alter outflow facility responses to cyclic AMP in cynomolgus monkeys. Exp. Eye Res..

[196] Ye M., Chen Z., Li M., Chen W., Zhang H., Wang J. (2019). Effect of topical application of adrenaline on Schlemm canal, trabecular meshwork and intraocular pressure. Medicine.

[197] Stamer W.D., Lei Y., Boussommier-Calleja A., Overby D.R., Ethier C.R. (2011). eNOS, a Pressure-Dependent Regulator of Intraocular Pressure. Investig. Opthalmol. Vis. Sci..

[198] Luo Z., Li M., Ye M., Ji P., Lou X., Huang J., Yao K., Zhao Y., Zhang H. (2020). Effect of Electrical Stimulation of Cervical Sympathetic Ganglia on Intraocular Pressure Regulation According to Different Circadian Rhythms in Rats. Investig. Opthalmol. Vis. Sci..

[199] Gallar J., Liu J.H. (1993). Stimulation of the cervical sympathetic nerves increases intraocular pressure. Investig. Ophthalmol. Vis. Sci..

[200] Alm A., Nilsson S.F. (2009). Uveoscleral outflow—A review. Exp. Eye Res..

[201] Johnson M., McLaren J.W., Overby D. (2016). Unconventional aqueous humor outflow: A review. Exp. Eye Res..

[202] Yücel Y.H., Johnston M.G., Ly T., Patel M., Drake B., Gumus E., Fraenkl S.A., Moore S., Tobbia D., Armstrong D. (2009). Identification of lymphatics in the ciliary body of the human eye: A novel “uveolymphatic” outflow pathway. Exp. Eye Res..

[203] Bill A. (1967). Effects of atropine and pilocarpine on aqueous humour dynamics in cynomolgus monkeys (*Macaca irus*). Exp. Eye Res..

[204] Bill A. (1969). Early effects of epinephrine on aqueous humor dynamics in vervet monkeys (*Cercopithecus ethiops*). Exp. Eye Res..

[205] Abaye D.A., Pullen F.S., Nielsen B.V. (2011). Practical considerations in analysing neuropeptides, calcitonin gene-related peptide and vasoactive intestinal peptide, by nano-electrospray ionisation and quadrupole time-of-flight mass spectrometry: Monitoring multiple protonations. Rapid Commun. Mass Spectrom..

[206] Selbach J.M., Schonfelder U., Funk R.H.W. (1998). Arteriovenous Anastomoses of the Episcleral Vasculature in the Rabbit and Rat Eye. J. Glaucoma.

[207] Goldmann H. (1951). Abflussdruck, Minutenvolumen und Widerstand der Kammerwasser-strömung des Menschen. Doc. Ophthalmol..

[208] Strohmaier C.A., Reitsamer H.A., Kiel J.W. (2013). Episcleral Venous Pressure and IOP Responses to Central Electrical Stimulation in the Rat. Investig. Opthalmol. Vis. Sci..

[209] Selbach J.M., Rohen J.W., Steuhl K.-P., Lütjen-Drecoll E. (2005). Angioarchitecture and Innervation of the Primate Anterior Episclera. Curr. Eye Res..

[210] Ladek A.M., Trost A., Bruckner D., Schroedl F., Kaser-Eichberger A., Lenzhofer M., Reitsamer H.A., Strohmaier C.A. (2019). Immunohistochemical Characterization of Neurotransmitters in the Episcleral Circulation in Rats. Investig. Opthalmol. Vis. Sci..

[211] Heermann S. (2017). Neuroanatomie der Sehbahn. Mon. Augenheilkd..

[212] May P.J., Warren S., Bohlen M.O., Barnerssoi M., Horn A.K.E. (2015). A central mesencephalic reticular formation projection to the Edinger-Westphal nuclei. Brain Struct. Funct..

[213] Motlagh M., Geetha R. (2021). Physiology, Accommodation. StatPearls.

[214] May P.J., Gamlin P.D. (2020). Is Primate Lens Accommodation Unilaterally or Bilaterally Controlled?. Investig. Opthalmol. Vis. Sci..

[215] Lara-Lacárcel F., Marín-Franch I., Fernández-Sánchez V., Riquelme-Nicolás R., López-Gil N. (2021). Objective changes in astigmatism during accommodation. Ophthalmic Physiol. Opt..

[216] Gamlin P.D., Yoon K. (2000). An area for vergence eye movement in primate frontal cortex. Nat. Cell Biol..

[217] Schor C.M., Lott L.A., Pope D., Graham A.D. (1999). Saccades reduce latency and increase velocity of ocular accommodation. Vis. Res..

[218] Domínguez-Vicent A., Monsálvez-Romín D., Del Águila-Carrasco A.J., Ferrer-Blasco T., Montés-Micó R. (2014). Changes in the anterior chamber during accommodation assessed with a Scheimpflug system. J. Cataract. Refract. Surg..

[219] Tigges M., Iuvone P.M., Fernandes A., Sugrue M.F., Mallorga P.J., Laties A.M., Stone R.A. (1999). Effects of Muscarinic Cholinergic Receptor Antagonists on Postnatal Eye Growth of Rhesus Monkeys. Optom. Vis. Sci..

[220] Schmid K.L., Wildsoet C.F. (2004). Inhibitory Effects of Apomorphine and Atropine and Their Combination on Myopia in Chicks. Optom. Vis. Sci..

[221] Huang J., Wen D., Wang Q., McAlinden C., Flitcroft I., Chen H., Saw S.M., Chen H., Bao F., Zhao Y. (2016). Efficacy Comparison of 16 Interventions for Myopia Control in Children. Ophthalmology.

[222] Barathi V., Beuerman R.W. (2011). Molecular mechanisms of muscarinic receptors in mouse scleral fibroblasts: Prior to and after induction of experimental myopia with atropine treatment. Mol. Vis..

[223] Barathi V.A., Weon S.R., Beuerman R.W. (2009). Expression of muscarinic receptors in human and mouse sclera and their role in the regulation of scleral fibroblasts proliferation. Mol. Vis..

[224] Lin H.-J., Wan L., Chen W.-C., Lin J.-M., Lin C.-J., Tsai F.-J. (2012). Muscarinic Acetylcholine Receptor 3 Is Dominant in Myopia Progression. Investig. Opthalmol. Vis. Sci..

[225] McBrien N.A., Stell W.K., Carr B. (2013). How does atropine exert its anti-myopia effects?. Ophthalmic Physiol. Opt..

[226] Abramson D.H., Franzen L.A., Coleman D.J. (1973). Pilocarpine in the Presbyope. Arch. Ophthalmol..

[227] Abramson D.H., Coleman D.J., Forbes M., Franzen L.A. (1972). Pilocarpine. Arch. Ophthalmol..

[228] Benozzi G., Cortina M.E., Gimeno E., Vantesone D.L., Solas A.E., Lorda G.M., Facal S., Leiro J., Orman B. (2021). A multicentric study of pharmacological treatment for presbyopia. Graefe’s Arch. Clin. Exp. Ophthalmol..

[229] Benozzi G., Perez C., Leiro J., Facal S., Orman B. (2020). Presbyopia Treatment with Eye Drops: An Eight Year Retrospective Study. Transl. Vis. Sci. Technol..

[230] Ono M., Takamura E., Shinozaki K., Tsumura T., Hamano T., Yagi Y., Tsubota K. (2004). Therapeutic effect of cevimeline on dry eye in patients with Sjögren’s syndrome: A randomized, double-blind clinical study. Am. J. Ophthalmol..

[231] Petrone D., Condemi J.J., Fife R., Gluck O., Cohen S., Dalgin P. (2002). A double-blind, randomized, placebo-controlled study of cevimeline in Sjögren’s syndrome patients with xerostomia and keratoconjunctivitis sicca. Arthritis Rheum..

[232] Ou K., Copland D.A., Theodoropoulou S., Mertsch S., Li Y., Liu J., Schrader S., Liu L., Dick A.D. (2020). Treatment of diabetic retinopathy through neuropeptide Y-mediated enhancement of neurovascular microenvironment. J. Cell. Mol. Med..

